# Ewing sarcoma of the great toe: a rare case report

**DOI:** 10.1097/RC9.0000000000000171

**Published:** 2026-02-11

**Authors:** Abdulaziz Alkhudhayri, Hamid Talal AlJohani, Abdulaziz Mohammed AlDakhil, Abdulrahman Bobseit, Abdullah A. Alhamdan

**Affiliations:** a General practitioner, Buraidah central hospital, Buraidah , Saudi Arabia; b Orthopedics Trauma and Foot and Ankle Fellow, Western University London Ontario, Canada; c Board-Certified Orthopaedic Surgeon, Prince Sultan Military Medical City, Riyadh, Saudi Arabia; d Head of Orthopedics Oncology Unit, King Faisal Specialist Hospital, Riyadh, Saudi Arabia; e Head of Orthopedics Oncology Unit, Orthopedics Department, Prince Sultan Military Medical City, Riyadh, Saudi Arabia

## Abstract

**Introduction and importance:**

Ewing sarcoma (ES) rarely arise in the toes, where nonspecific pain and swelling can mimic benign conditions, delaying diagnosis. Early recognition is critical because modern multimodal therapy can achieve limb preservation and high rates of disease control even at unconventional sites.

**Case presentation:**

A healthy 22-year-old man developed progressive pain and swelling of the left hallux after partial nail excision for a presumed ingrown toenail. The wound failed to heal, evolving into a friable 3 × 3 cm dorsal mass. Radiography and MRI demonstrated an aggressive lytic lesion destroying the distal phalanx with soft-tissue extension. Incisional biopsy revealed small round blue cells strongly positive for CD99, and fluorescence in situ hybridization confirmed an EWSR1 rearrangement consistent with ES. Staging PET-CT showed no metastases. The patient received six cycles of vincristine–doxorubicin–cyclophosphamide alternating with ifosfamide–etoposide, followed by partial amputation of the distal phalanx with clear margins. Histology showed 98% tumor necrosis. Adjuvant chemotherapy was completed uneventfully. At 12-month surveillance, MRI and PET-CT showed no local recurrence or distant spread, and the patient had painless, functional ambulation.

**Clinical discussion:**

This case highlights three key lessons: 1. *Persistent or atypical digital lesions warrant oncologic evaluation.* Soft-tissue masses that do not resolve after routine care should prompt advanced imaging and biopsy. 2. *Molecular confirmation is pivotal.* Detection of an EWSR1-FLI1 family fusion secures the diagnosis and guides therapy. 3. *Neoadjuvant chemotherapy enables limb-sparing surgery.* Pre-operative tumor shrinkage facilitated margin-negative resection while preserving toe function, and a 98% necrosis rate predicts favorable prognosis. Vigilant, long-term surveillance remains essential because ES can relapse late.

**Conclusion:**

Ewing sarcoma of the great toe is exceptionally rare but can be successfully treated when identified early and managed with coordinated multimodal therapy. Clinicians should maintain vigilance for malignancy in non-healing digital lesions to ensure timely intervention and durable outcomes.

## Introduction

Ewing sarcoma is a rare and aggressive malignancy that affects bones and surrounding soft tissue^[^1^]^. This condition is commonly seen in children and young adults, and accounts for about 1% of all childhood cancers; in fact, Gargallo *et al* estimated about 1.2 cases out of every 1 million people as incidence in the USA^[^1^]^. Ewing sarcoma is characterized by small and round blue cells and its association with specific chromosomal translocations, most commonly the EWSR1-FLI1 fusion gene, which plays a critical role in diagnosis^[^1,2^]^. The condition often presents with localized pain and swelling, alongside occasional fevers and weight loss^[^3^]^.

HIGHLIGHTS

- Multimodal work-up (X-ray, MRI, histopathology, FISH) confirmed EWSR1 rearrangement and guided limb-sparing surgery.
- Neoadjuvant vincristine–doxorubicin–cyclophosphamide/ifosfamide–etoposide achieved 98% tumor necrosis.
- Function-preserving partial amputation plus adjuvant chemotherapy yielded recurrence-free survival at 12 months.
- Case underscores the need to biopsy persistent toe lesions in young adults and illustrates current best practice for rare acral Ewing sarcoma.

Treating Ewing sarcoma mostly involves a multimodal approach of chemotherapy, surgery, and at times, radiotherapy, whose goal is to achieve local and systemic control^[^3–5^]^. Technological advancements in imaging and molecular diagnostics have enhanced our capacity to detect and characterize Ewing sarcoma, which can improve patient outcomes upon timely diagnosis. Given the rarity of distal phalanx involvement and its frequent misdiagnosis as a benign condition, we report this case to highlight diagnostic pitfalls, the role of EWSR1 testing, and limb-sparing management, in accordance with the SCARE checklist^[^6^]^.

## Case presentation

The patient is a medically free 22-year-old male, who presented with a 2–month history of progressive swelling and pain in his left big toe that was associated with minimal blood discharge but no pus or systemic symptoms such as fever. The patient’s medical history was unremarkable and had only presented with an umbilical hernia that was repaired at the age of 6 years, with no other significant past medical or family history.

The patient underwent a partial ingrown nail excision at another hospital about a month prior to presentation, where the wound was sutured. However, a hematoma developed immediately after suturing, necessitating the removal of the sutures on the same day. Subsequently, the wound failed to heal and progressed to an ulcer with discharge, upon which the patient became concerned, and sought a second medical opinion. The patient thereafter came for an examination at our medical facility. He was alert and oriented, with his left big toe notably swollen and congested, hypertrophied skin over the nail bed and a firm 3 × 3 cm mass on the dorsal aspect. The mass bled easily, was non-necrotic and exhibited no active discharge. Peripheral pulses were intact, and no neurovascular deficits were present.

**Figure 1.**
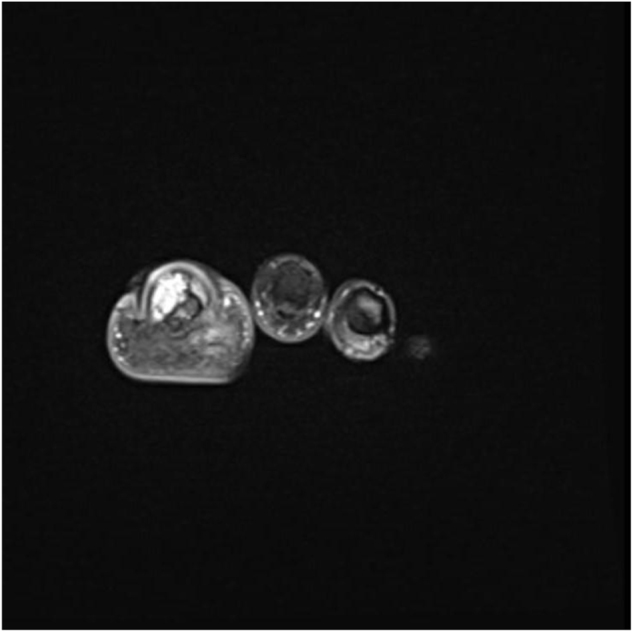
MRI of the left hallux. (a) Coronal T2-weighted image demonstrates a hyperintense soft-tissue component adjacent to the distal phalanx. (b) Sagittal T2-weighted image confirms the high-signal lesion with associated soft-tissue component.

**Figure 2.**
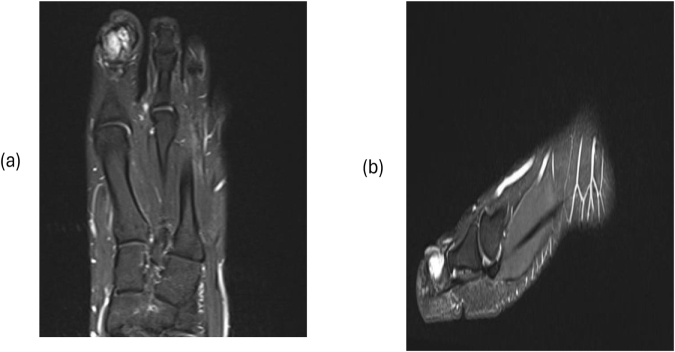
Anteroposterior X-ray of the left foot revealing an irregular and destructive lesion involving the distal phalanx of the first toe.

**Figure 3.**
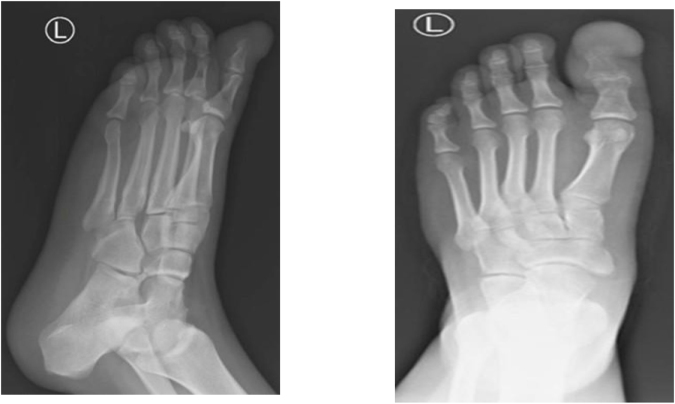
Sagittal MRI of the left foot revealing a heterogeneously enhancing soft tissue mass centered on the distal phalanx of the first toe.

**Figure 4.**
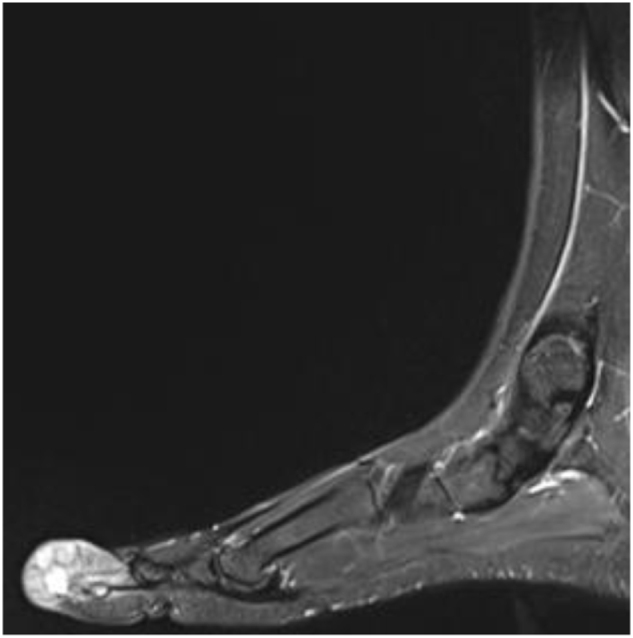
Axial T1-weighted MRI of the left hallux showing a low-signal lesion in the distal phalanx with an associated soft-tissue component.

**Figure 5.**
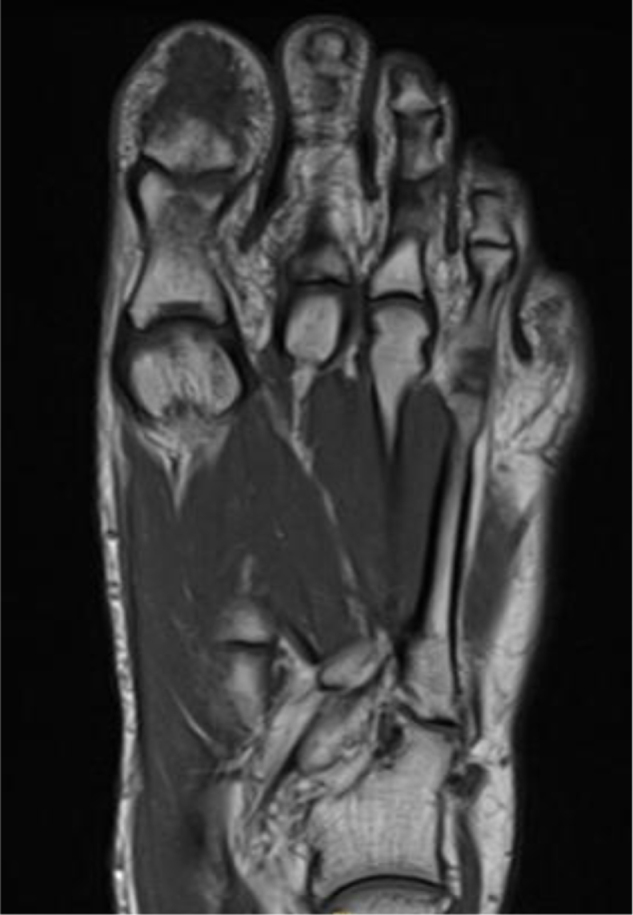
Coronal MRI of the left foot demonstrates a heterogeneously enhancing mass centered around the distal phalanx of the first toe.

Laboratory findings were barely conclusive, whereby stable values for white blood cells, hemoglobin, and platelets over several days were observed with no evidence of systemic infection. However, radiographic imaging revealed a soft tissue mass with superficial calcifications and periosteal reaction at the distal phalanx. The patient was subsequently examined under MRI (T1, STIR, and post-contrast sequences), which demonstrated an aggressive destructive marrow-replacing lesion of the distal phalanx of the great toe with cortical destruction and a dorsal extraosseous soft-tissue component extending into the subcutaneous fat with an exophytic skin component. The lesion measured 4.4 × 2.9 × 3.0 cm, was low signal on T1, high on STIR with bubbly cystic changes and internal septations, and showed avid homogeneous enhancement on post-contrast images, although artifact obscured much of the lesion. Overall, these findings suggested an aggressive lesion with both bone and soft tissue involvement, and a giant cell tumor was considered in the initial differential (Figs 1–5).

**Figure 6.**
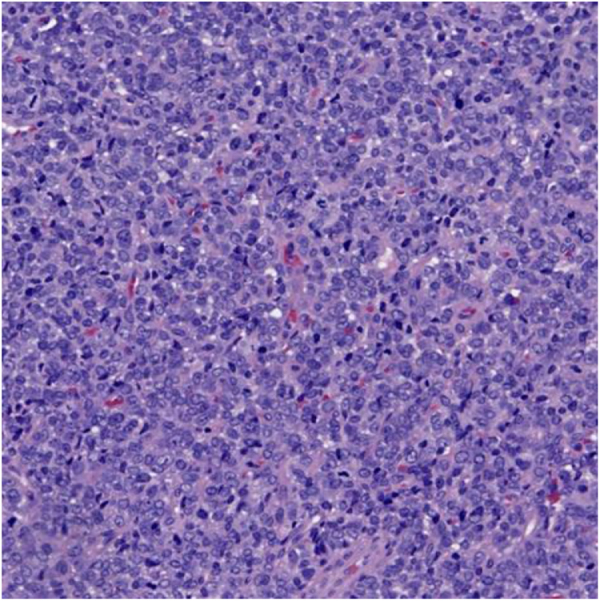
High-magnification hematoxylin and eosin (H&E) stained section of Ewing sarcoma.

**Figure 7.**
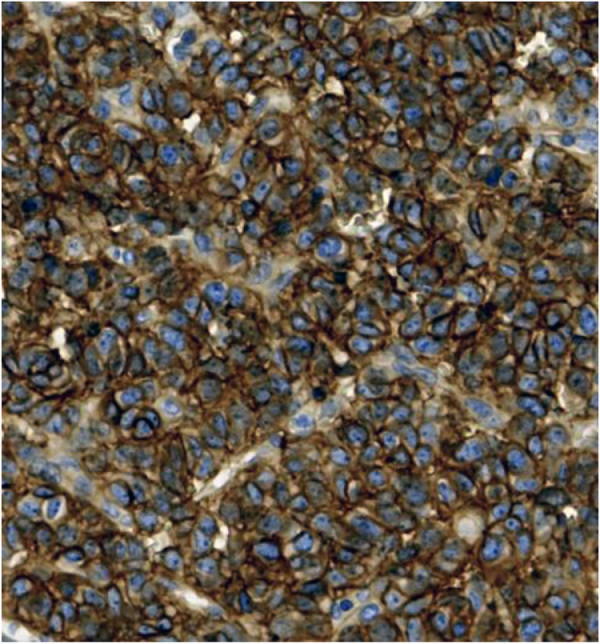
Immunohistochemical staining for CD99.

The imaging results necessitated a biopsy, which confirmed Ewing sarcoma through histopathological and immunohistochemical staining, and *FISH* analysis, which identified the EWSR1 rearrangement (Figs 6 and 7). Post-treatment FDG PET-CT performed after hallux amputation and chemotherapy showed no focal FDG uptake at the operative site and no regional or distant FDG-avid lesions, indicating no evidence of local recurrence or metastatic disease. The liver reference SUVmax was 3.3 (previously 3.0), and chest CT revealed localized disease without distant metastasis, as shown by immunohistochemical staining for CD99 in Figure 7 below, which demonstrated strong, diffuse membranous positivity in tumor cells, a characteristic feature of Ewing sarcoma.

Treatment began with neoadjuvant chemotherapy and then followed by surgical excision. Partial amputation of the distal phalanx was done during the initial surgery to achieve complete resection of the tumor while preserving function. Adjuvant chemotherapy was then administered postoperatively, and it demonstrated a significant tumor response with 98% necrosis on histopathology. Subsequent surveillance imaging over several months showed no signs of tumor recurrence or metastatic disease. The patient remains under regular multidisciplinary follow-up.

## Discussion

The most frequent histologic types include Ewing sarcoma, osteosarcoma, and chondrosarcoma^[^7^]^. Management typically involves early diagnosis and coordinated multidisciplinary care with either wide local excision or partial amputation to achieve negative margins, combined with systemic chemotherapy in chemosensitive tumors such as Ewing sarcoma^[^5^]^.

The atypical location of the tumor, as seen in this case, poses diagnostic and therapeutic challenges that require a multimodal approach characterized by a high index of suspicion. Due to the initial presentation of pain, swelling, and a mass localized to the left big toe, it was initially misattributed to an ingrown toenail and associated complications. The clinical and radiological findings bring to us several differential diagnoses, including osteomyelitis, giant cell tumors, and other benign or inflammatory toe lesions. Chronic osteomyelitis can present with pain, swelling, and lytic changes in the phalanx, but the absence of systemic symptoms, inflammatory markers, made infection unlikely. Giant cell tumors were also suspected radiographically because of the expanded lytic appearance however, the lesion’s permeative margins and soft-tissue extension were atypical for GCT. Other differential possibilities, such as enchondroma, subungual exostosis, or epidermal inclusion cyst, were excluded based on the aggressive pattern of bone destruction and MRI signal characteristics. Definitive histopathology showing uniform small round blue cells and immunohistochemistry positive for CD99, together with fluorescence in-situ hybridization confirming EWSR1 rearrangement, conclusively established the diagnosis of Ewing sarcoma.

We acknowledge the important role that anatomical imaging modalities play in the diagnostic process. Plain radiographs revealed periosteal reaction and soft-tissue calcification, and MRI provided detailed visualization of the lesion’s extent. According to Guimarães *et al*, anatomical imaging methods play a crucial role in determining the extent of the primary tumor, compensating for the limitations of PET/CT tools during staging and restaging, and assessing the patient’s therapeutic response^[^8^]^. Assessment of tumors in patients should comprise of a combination of anatomical imaging techniques and PET/CT tools^[^8–10^]^.

A similar case was described by Dunn *et al* in 1976 involving Ewing sarcoma of the great toe, the case highlighted a myriad of diagnostic challenges posed by this rare presentation^[^11^]^. Similar to our case, the tumor’s location outside the pelvis and long bones initially led to misdiagnosis, which necessitated a biopsy in indolent or refractory lesions presumed to be infectious. However, given that this is possibly among the very first documented cases of Ewing sarcoma, they used Technetium Polyphosphate as a diagnostic tool, which has since been replaced by advanced imaging modalities such as MRI and PET-CT. Different from our case, the patient had metastasis, and vigorous chemotherapy and radiotherapy had limited efficacy. Early diagnosis, neoadjuvant chemotherapy, and surgical intervention led to a favorable outcome without metastasis in our case. The apparent difference in outcome underscores the importance of timely intervention and modern treatment protocols in improving the prognosis for rare distal presentations of Ewing sarcoma.

Hagihara *et al* reported a patient with Ewing sarcoma presenting with very late metastasis to the skull, 21 years after the initial diagnosis and treatment^[^12^]^. Both this case and ours emphasize the unpredictable nature of Ewing sarcoma’s clinical course. Hagihara *et al* present a very late recurrence or metastasis and a prolonged period in which the patient was disease-free which goes to show how important extended surveillance is for Ewing sarcoma patients. The two cases, emphasis lies on the need to aggressively treat the condition with chemotherapy and surgical intervention to prevent metastasis and disease spread. Primary malignant bone tumors of the foot are extremely rare when compared with other skeletal malignancies,

Another case report by Morlote *et al* detailed a rare adamantinoma-like variant of Ewing sarcoma in the thyroid gland^[^13^]^. This case involved a 20-year-old woman with a thyroid tumor showing classic EWSR1 rearrangement but with epithelial differentiation. The case highlights the diverse histological and immunohistochemical presentations of Ewing sarcoma. Distant from our case, the adamantinoma-like variant is a testament to the tumor’s ability to arise in unusual non-osseous locations, complicating diagnosis. However, we note that advanced molecular diagnostics and imaging techniques are required to confirm the EWSR1 gene rearrangement. Regardless of location, accurate genetic testing using *RT-PCR* and *FISH* tools is important in achieving an accurate classification of Ewing sarcoma upon diagnosis. A notable similarity is the multimodal treatment strategy of chemotherapy and surgery that was adapted to the unique tumor characteristics and locations.

Lastly, we evaluated the case report by Grassetti *et al* which was another unusual presentation of Ewing sarcoma as a primary cutaneous lesion on the foot^[^14^]^. The case involved a large mass measuring 9.5 × 8 cm without bone involvement, which is different from our case, in which bone destruction necessitated amputation. In comparison, this emphasizes the rarity of primary cutaneous Ewing sarcoma and its resemblance to other cutaneous tumors, which poses significant diagnostic challenges. The patient was treated successfully with surgical excision alone without the need for aggressive multimodal treatment, which was used to limit the tumor’s invasive nature and bone involvement in our case. The broad spectrum of clinical behavior in Ewing sarcoma is clear, and the importance of individualized treatment strategies based on tumor characteristics and location cannot be emphasized enough.

## Conclusion

Toe Ewing sarcoma is rare but potentially curable with timely diagnosis and coordinated multimodal therapy. Clinicians should consider malignancy in non-healing digital lesions to enable limb-sparing treatment and durable disease control. Given the risk of late recurrence or metastasis, long-term multidisciplinary follow-up is strongly recommended for all patients with Ewing sarcoma, even after initial remission.

## Data Availability

The data that support the findings of this study are available from the corresponding author upon reasonable request.
